# Case of Facial Neuralgia, Arising from External Violence, Treated with Success by Division of the Affected Nerves

**Published:** 1820-06

**Authors:** 

**Affiliations:** Saint-Valery-sur-Somme.


FOR THE LONDON MEDICAL AND PHYSICAL JOURNAL.

***Case of Facial Neuralgia, arising from external Violence, treated with success by Division of the affected Nerves.*** **By Dr. Ravin, of Saint-Valery-sur-Somme.**

THOMAS GOMBERT, fifty-three years of age, of a thin but robust habit of body, and moderate stature, struck his head with considerable violence against a large table: the blow occurred on the right side of the head; he fell down on the receipt of it, and remained insensible for several minutes. He suffered pains in his head for several days afterwards; but he nevertheless exposed himself continually to the weather in a very changeful season. The pains at length ceased, and he was two months without suffering any uneasiness from the accident; but at the end of this time he suddenly experienced very acute pain, which appeared to arise at the part where he received the blow, and extend thence along the forehead and the whole of the right cheek. The whole of this side of the face was affected with convulsive motions; the patient made horrible grimaces, and cried aloud, saying, that it seemed as if his eye and face were being torn to pieces with hooks. This returned in paroxysms, at first about once, twice, or thrice a-day. His sleep at night was also much disturbed. They soon became so violent and insupportable, that the patient was obliged to cease his ordinary occupations. The convulsions recurred almost every instant; he could neither speak, eat, nor sleep. When he attempted to chew, the pain was much increased, or a paroxysm immediately produced in case of its being absent.

Some deep-seated carious affection of the jaw was suspected to exist; and all the upper teeth on the right side were drawn in succession, although none of them were diseased, but without advantage. Purgatives, bleedings, and embrocations, were employed without benefit. He remained for two years in this deplorable state, when he was brought to me almost in spite of himself, for he had no hope of obtaining relief. He walked almost double, came into my presence in tears, and could not speak a word. The foregoing account was then given me by his wife. He listened to what I had to say on his case with great anxiety; and, on my expressing expectations of a cure, he determined to submit to any means I chose to resort to.

I determined to divide the frontal branch of the ophthalmic nerve, which was done on the following day, the 28th June, 1819, just as it passed above the superciliary arcade. The patient felt instant ease: he said, it seemed as if the pain went out of the divided part. The convulsions which afterwards occurred were less painful, but they nevertheless occurred as frequently as before. I let him remain in this state for two days, that I might examine him more at leisure. He was fed with soup, as he had been for some time previously.

I discovered that the pain no longer sprang from the same spot, neither did it run along the frontal nerve: instead of commencing on the head, at the middle of the parietal bone, it commenced on the forehead, then descended outwardly towards the point of the eye-brow, whence it extended to the eye-lid and the cheek. As, by the section of the frontal nerve, less nervous filaments were in action during the paroxysm, it was easy to understand why the convulsions and pains were less violent than before the division of that nerve; and the eye-lids, besides, were less forcibly pressed on the globe of the eye. I did not despair of obtaining perfect success; and the courage of the patient, roused by the relief he had already experienced, determined him to submit to as many operations as I should propose to perform.

On the 1st of July, in the morning, I made a long incision from the nose to the temple; I cut through all the soft parts which lay between the bone and my bistoury, and thus destroyed all the nervous communications which could in this part give rise to pain: it entirely ceased on this being effected. The patient felt it go away, as on the first day. I attached the eye-lid to the integuments of the forehead by two sutures, and covered the wound with a pear-tree leaf, because these leaves are smooth and concave: the eye-lid was thus suspended in its proper situation. This dressing was covered by a compress and a handkerchief. I dressed the wound twice a-day. The leaf fell off of itself, which pledgets of lint will not do; the purulent matter which issued from the wound remained fluid under the leaf, and did not dry on the edges of the incision: besides these advantages from the use of the leaf, it was incapable of irritating the wound, as even the finest lint always does in some degree.

The patient had no fever; nevertheless, I ordered very spare diet for three days.

On the day after this operation he slept with the greatest calmness, and awoke without feeling any pain: his joy was inexpressible. But on the third day he had some uneasiness, not violent, it is true, but sufficient to cause him to make a grimace now and then, and casually to make him stop when he was taking food. On this day I had persuaded him to try to eat a little bread. These slight pains were produced by some degree of inflammatory tumefaction of the parts, and by the ligatures which irritated the skin. The muscles of these parts were so sensible, that the slightest irritation of their nerves set them all into action. Three days afterwards, when the inflammation had diminished, the pains became more or less slight; and five days afterwards, when I removed the ligatures, they totally disappeared. The wound, as well as the disease, was cured in ten days.

The patient sat out at the end of a month for Louroi, able to speak, eat, and sleep, as well as before his accident, and suffering no uneasiness whatever. Eight months have since elapsed, and he continues in health, having never experienced the slightest recurrence of pain.—*Journal Universel des Sciences Medicales, Avril* 1820.

